# A Survey of the Menstrual Status of Female College Students

**DOI:** 10.3390/healthcare11081108

**Published:** 2023-04-12

**Authors:** Maki Maekawa, Aya Miyamoto, Hiromi Ariyoshi, Koji Miura

**Affiliations:** 1 Department of Physical Education, International Pacific University, 721 Kanonji, Seto-cho, Higashi-ku, Okayama 709-0863, Japan; 2 Ariyoshi Occupational Health Consultant Office, Fukuoka 815-0038, Japan

**Keywords:** menstruation-associated symptoms, youth, menstrual education, women’s health, wellbeing

## Abstract

Education about menstruation is a sensitive topic for young female students; providing appropriate knowledge is essential for maintaining and improving their health. The present study was conducted to collect data corresponding to different factors affecting health among young individuals; the menstrual status, exercise habits, sleep status, and body composition of these individuals as well as the relationships among these factors, were evaluated. Altogether, 200 female students responded to the survey; 129 completed all the physical measurement items. As a case study, face-to-face interviews regarding menstrual symptoms were conducted. Results showed that 49/200 (25%) and 120/200 (60%) participants experienced moderate or severe pain before and during menstruation, respectively. The degree of pain one week before menstruation and during menstruation were significantly positively correlated (r = 0.573, *p* < 0.01). When analyzed as group data, it was difficult to identify the relationship between menstrual status, exercise habits, and sleep status; these were found to be intricately associated with various factors. The case study analysis confirmed that some individuals experienced physical and psychological symptoms, such as irregular menstrual cycles, premenstrual syndrome, and severe menstrual cramps.

## 1. Introduction

Women experience various unique health issues specific to them owing to the effects of female hormones, physical and mental changes associated with different life stages, and other factors. Female hormones are regulated by a gonadotropin-releasing hormone secreted by the hypothalamus that acts on the pituitary gland, gonadotropin–stimulating hormones (follicle–stimulating and luteinizing hormones) secreted by the pituitary gland that acts on the ovaries, and the hormones estrogen and progesterone secreted by the ovaries; the feedback mechanisms of these hormones also play a role in their regulation. Hormone secretion, menstrual cycle, and menstruation-associated symptoms are influenced by stress on the body and mind as well as by lifestyle factors such as sleep and eating habits [1,2,3,4,5,6]. The maintenance of the menstrual cycle and the severity of symptoms are directly related to the daily life of the individuals.

Changes in female hormones associated with the menstrual cycle cause various physical and mental symptoms–referred to as menstruation-associated symptoms–particularly in women during pubescent and sexual maturity. Among these symptoms, those not involving organic factors are broadly classified into functional dysmenorrhea, which occurs during the menstrual phase, and premenstrual syndrome (PMS) and premenstrual dysphoric disorder (PMDD), which occur during the luteal phase. Risk factors for dysmenorrhea include the age of <20 years, attempts to achieve weight loss, depression and anxiety, disruption of social networks, heavy menses, nulliparity, and smoking [7]. These risk factors also affect girls at a young age. The severity of PMS and PMDD (regarded as a severe form of PMS) in adolescents is reportedly significantly higher than that in adults [8,9]. Symptoms are caused by fluctuations in female hormones during the normal ovulatory cycle and are cyclically repeated, thereby adversely affecting the quality of life of these individuals. Menstrual symptoms are often overlooked because they are not directly life-threatening; however, they are a highly prevalent issue, and their severity has been reported to interfere with studies, school attendance, and overall performance [10,11,12].

The hormone leptin is important for the maintenance of normal menstruation during puberty. Leptin, produced in adipocytes, acts on the hypothalamic satiety center to suppress appetite and is involved in weight regulation and energy balance. Moreover, leptin plays a major role in activating the hypothalamic–pituitary–ovarian system. Leptin secreted by increased body fat stimulates the secretion of luteinizing hormone-releasing hormone from the hypothalamus, which increases gonadotropin secretion and stimulates the ovaries. The subsequent secretion of estrogen and progesterone causes the appearance of secondary sexual characteristics [13,14,15]. Previous studies have demonstrated an association between menstrual irregularities and body mass during puberty and sexual maturity [16,17,18]. Abnormal menstruation in underweight individuals can be attributed to a decrease in leptin, which is reportedly influenced by a decrease in body fat owing to weight loss [19,20]. Conversely, abnormal menstruation in overweight and obese individuals involves an increase in leptin that affects ovarian cells and hormone secretion [15,21,22].

Because estrogen possesses bone-strengthening properties, abnormal menstruation during puberty affects the acquisition of bone mass. Bone mass is considered to peak around the age of 20 years [23,24]; sufficient bone mass should be acquired during the period from puberty to the age of 20 years. However, if estrogen secretion declines for a long period during this interval owing to amenorrhea or irregular menstruation, acquiring sufficient bone mass becomes challenging. This increases the risk of osteoporosis and fractures during puberty and maturation as well as during the later stages of life. Therefore, the acquisition of appropriate knowledge about menstruation is important for the maintenance and improvement of the health of young individuals.

In 1992, the American College of Sports Medicine (ACSM) cautioned about three common health issues in female athletes–disordered eating, amenorrhea, and osteoporosis–describing them as the “female athlete triad (FAT).” Following a revision in 2007, the ACSM now recommends the importance of prevention and management as “low energy availability (with or without eating disorders),” “hypothalamic amenorrhea,” and “osteoporosis” in female athletes [25,26,27]. Energy deficiency negatively affects menstrual function and bone health and influences several other aspects of growth and development (mental, cardiovascular, digestive, immune, endocrine, and metabolic systems), thereby resulting in poor performance. To spread awareness about broader health issues in athletes, the International Olympic Committee published a consensus statement in 2014 called “Beyond the Female Athlete Triad: Relative Energy Deficiency in Sport (RED-S)” [28,29]. Considering these recommendations and announcements, the “White Paper on Gender Equality” published in 2018 by the Cabinet Office of Japan stated that recognizing the issues faced by female athletes, including the FAT, and providing appropriate support will have beneficial implications for the advancement of women in society as a whole and not solely in the field of sports [30]. In addition, the Sports Agency of Japan has been implementing measures to promote women’s participation and success in society by creating an environment that encourages women’s participation in playing, watching, and supporting sports [31].

However, education and knowledge of health issues experienced by women, including menstruation, remain far from reaching young individuals and those who are involved in their lives. In a recent study conducted from January 2017 to May 2019, Logue et al. (2020) summarized the prevalence of low energy availability in various sports activity groups; the estimated prevalence rates ranged from 22% to 58% [32]. In addition, a recent cross-sectional survey of head athletic trainers examining their knowledge of the FAT and RED-S conducted at National Collegiate Athletic Association member institutions reported that nearly all trainers (98.61%, *n* = 281) were aware of the FAT, but only one-third (32.98%, *n* = 94) were aware of RED-S [33]. Furthermore, Ebina et al. (2023) conducted a survey of Japanese high school health and physical education teachers on their attitudes toward menstruation and its associated issues; they found that >50% of the male teachers surveyed did not possess a sufficient understanding of amenorrhea, osteoporosis, and the FAT [34]. Moreover, at the authors’ affiliated university, there are many consultations from students on menstruation, such as irregular menstruation, menstrual cramps, and their impact on daily life and exercise. However, these women themselves also lack an understanding of the menstrual cycle, appropriate management and understanding of PMS, menstrual cramps, and irregular menstruation, as well as knowledge about the FAT and RED-S.

Therefore, the present study was conducted to collect data for investigating possible measures for education and health management of women’s health issues, including menstruation, in a young age group. Further, the conditions of female college students, including menstrual status, exercise habits, sleep status, and body composition, were surveyed to evaluate possible causal differences in the degree of menstrual pain.

## 2. Materials and Methods

### 2.1. Participants

A questionnaire survey on menstrual status, exercise habits, sleep status, and body composition measurements was conducted among female students affiliated with K University. The purpose of the survey was explained to the participants; responses were obtained from those who gave their consent. Altogether, 200 individuals responded to the questionnaire survey. Of these, 129 completed all physical measurements. This study was conducted after obtaining approval from the ethics committee of the International Pacific University (approval no: 20-014).

### 2.2. Questionnaire Survey

The participants were asked to respond to a web-based questionnaire. The survey period was from 21 July to 18 August 2021. The survey questions were divided into the following categories: (1) menstrual status, (2) exercise habits, and (3) sleep status.

#### 2.2.1. Menstrual Status

Participants were retrospectively asked to indicate their menstrual cycle, the number of days of menstrual bleeding, and the degree of pain one week before and during menstruation in the last three months. For the questions on the degree of pain, a visual analog scale was used; the participants responded with the number most suitable on an 11-point scale ranging from 0 (no pain at all) to 10 (the most severe pain imaginable).

#### 2.2.2. Exercise Habits

Participants also listed their specializations in sports, years of competition, level of competition, the average number of practice or training days per week (training frequency), and the average number of practice or training hours per day (training duration).

#### 2.2.3. Sleep Status

The Japanese version of the Pittsburgh Sleep Quality Index (PSQI) was used to assess sleep quality [35]. This questionnaire was designed to assess the following: C1: sleep quality (subjective evaluation of overall sleep), C2: time to fall asleep (ease of falling asleep after going to bed), C3: sleep duration (length of total sleep time), C4: sleep efficiency (ratio of actual sleep time to time in bed), C5: sleep difficulty (evaluation of the frequency of waking up in the middle of the night), C6: sleep medication use (evaluation of the frequency of medication use to fall asleep), and C7: difficulty staying awake during the day (evaluation of daytime sleepiness and depressed mood associated with sleep issues). Each item was rated on a four-point scale from 0 to 3, with a higher total score (out of 21) indicating greater sleep disturbance, i.e., worse sleep quality. A total score of ≥6 was considered as having poor sleep quality [36]. Participants were asked to answer the questionnaire retrospectively based on their sleep status in the past month.

### 2.3. Body Measurements

Whole body volume, whole body surface area, and the length and circumference of each body part were measured using a three-dimensional human body measurement device, Bodyline Scanner, (SYMBOL, Tokyo, Japan). Body weight, fat mass, fat-free mass, and body fat percentage were measured using the InBody 430 (InBody Japan, Tokyo, Japan). Body composition was evaluated using the InBody Score and Skeletal Muscle Index. Bone age was determined using un ultrasonic bone mass measuring device, Benus evo (Nihon Kohden Corporation, Tokyo, Japan).

### 2.4. Statistical Analysis

In the questionnaire survey, a simple tabulation of the respondents (*n* = 200) was performed. Pearson’s product–rate correlation coefficients were obtained for each of the questionnaire items to confirm correlations. Participants who responded to both the questionnaire and physical measurements (*n* = 129) were grouped according to the degree of pain one week before and during menstruation; comparisons were tested with a one-way analysis of variance, with Bonferroni correction used for subsequent testing. To examine differences in the degree of pain before and during menstruation based on the level of physical activity, participants were classified into two groups: those who played competitive sports (athlete group) and those who were involved in athletic club activities several times a week or did not belong to any athletic club (non-athlete group). The classification of pain severity was based on a scale of 0 for no pain at all to 10 for the most severe pain imaginable; participants responding 0 were classified as the no pain group, 1–4 as the mild group, 5–7 as the moderate group, and 8–10 as the severe group. All statistical analyses were performed using SPSS 26 (IBM Japan, Tokyo, Japan), with the significance level set at <5%.

### 2.5. Case Study

Personalized interviews were conducted with participants regarding the type and severity of their menstrual symptoms and other physical or mental concerns. The interviews were conducted in a quiet room. Participants and interviewers were given numerous opportunities to talk with each other prior to the official interview, fostering a relationship of mutual trust.

## 3. Results

### 3.1. Questionnaire Survey on Conditions Related to Menstrual Status, Exercise Habits, and Sleep Status (n = 200)

#### 3.1.1. Menstrual Status

The mean and standard deviation of the participant’s age, height, and weight were 18.7 ± 1.0 years, 158.5 ± 6.5 cm, and 56.8 ± 9.0 kg, respectively. Regarding menstrual cycles, 152 (76%) participants reported constant cycles, 47 (23.5%) reported irregular cycles, and 1 (0.5%) reported no menstruation for >3 months. The number of days of menstrual bleeding was ≤3 days for 24 (12.0%), 4–6 days for 162 (81%), and >7 days for 14 (7.0%). Regarding menstrual pain one week before and during menstruation, ratings ranged from 0 for no pain at all to 10 for the most severe pain imaginable. Further, before and during menstruation, 80 (40%) and 14 (7%) participants reported no pain (choosing 0), 71 (36%) and 66 (33%) reported mild pain (choosing 1–4), 35 (18%) and 60 (30%) reported moderate pain (choosing 5–7), and 14 (7%) and 60 (30%) reported severe pain (choosing 8–10), respectively (Table 1). There was a significant positive correlation between the degree of pain one week before menstruation and the degree of pain during menstruation (r = 0.573, *p* < 0.01).

#### 3.1.2. Exercise Habits

Table 2 shows the sports specializations of the participants. The number of years of competition was 7.7 ± 4.4 years. Further, 84 (42.0%) participants were national-level athletes, 62 (31.0%) were regional-level athletes, and 51 (25.5%) were athletes of other levels; 3 (1.5%) participants did not respond to this question. Of the respondents, 31 (15.5%) belonged to athletic club activities, and 33 (16.5%) had no affiliation.

The average training frequency was 4.2 ± 2.4 days, and the average training duration was 2.6 ± 1.6 h. Training frequency and training duration were significantly positively correlated (r = 0.754, *p* < 0.01).

#### 3.1.3. Sleep Status

The mean value of the Japanese version of the PSQI obtained in this study was 4.5 ± 2.8 points. Of the respondents, 143 (71.5%) exhibited a PSQI of <6 points, and 57 (28.5%) had a PSQI of ≥6 points, indicating poor sleep quality. Sleep duration for the poor sleep quality group (PSQI ≥ 6 points) averaged 6.2 ± 1.0 h; 6 respondents (11%) slept for ≤5 h, 28 (49%) slept for 5–6 h, and 23 (40%) slept for >6 h. Sleep duration for those with a PSQI of <6 points averaged 6.1 ± 1.1 h; 10 (7%) respondents slept for ≤5 h, 62 (43%) for 5–6 h, and 71 (50%) for >6 h. Those with a PSQI of ≥6 points required a longer time to fall asleep after going to bed and had a lower wakefulness status during the day compared with those with a PSQI of <6 points (Table 3).

### 3.2. Differences in Physical Condition and Menstrual Pain Symptoms (n = 129)

#### 3.2.1. Pain and Condition One Week before Menstruation

When classifying the group according to pain one week before menstruation, 56 women were included in the no-pain group, 43 in the mild pain group, 24 in the moderate pain group, and 6 in the severe pain group (Table 4). A difference between the groups was observed in terms of the InBody Score; the score was significantly lower in participants with severe pain than in those without pain [F (3, 125) = 3.50, *p* < 0.05]. Moreover, a significant negative correlation was observed between pain one week before menstruation and InBody Score (r = −0.276, *p* < 0.01), with participants with more premenstrual pain having a lower InBody Score. The InBody Score is an original classification system developed by InBody Japan Co., Ltd. to facilitate an easier understanding of body composition measurements. The score represents the estimated appropriate values for muscle mass and body fat relative to the appropriate body weight. The standard score is 80 points, and the score is lowered if the person’s body composition is low in muscle mass and high or low in fat mass relative to the appropriate body weight. A high score indicates high muscle mass and normal body fat content, whereas a low score indicates an imbalance between muscle and fat. An InBody Score of <70 is defined as frail/obese, 70–80 as general, 80–90 as healthy, and ≥90 as muscular. For lean body mass, compared with the mild pain group, the no-pain group showed a trend toward greater muscle mass (*p* = 0.088). Body fat percentage tended to be higher in the severe pain group than in the no-pain and mild-pain groups (*p* = 0.096 for no pain vs. severe pain, *p* = 0.088 for mild pain vs. severe pain). No significant differences or associations were observed between the pain intensity and sleep patterns in terms of PSQI total score or sleep duration.

#### 3.2.2. Pain and Condition during Menstruation

When classifying the group according to pain during menstruation, 11 participants were categorized in the no-pain group, 42 in the mild-pain group, 37 in the moderate-pain group, and 39 in the severe-pain group. All groups showed no significant differences in all items.

### 3.3. Individual Cases Regarding Menstruation-Associated Symptoms

#### 3.3.1. Case 1

A 20-year-old woman with a body mass index (BMI) of 19.3 kg/m^2^ was examined. She was a member of a track and field team. Her major concern was that her menstrual cycle had become irregular following a significant weight loss of 3–4 kg and an increase in practice during her high school years. In addition, her menstruation stopped for more than 3 months during her senior year of high school. Although she had no additional significant weight gain or loss, her menstrual cycle remained irregular after entering college. She had never consulted a gynecologist before and was planning on visiting one but felt hesitant to consult a male gynecologist. She also mentioned that she would not hesitate to visit a female gynecologist.

#### 3.3.2. Case 2

A 19-year-old woman with a BMI of 21.2 kg/m^2^ was examined. She was a member of a track and field team. She reported abdominal pain and headache before and during menstruation, which interfered with her daily life. As a result, she could not attend her classes or exercise during menstruation and experienced depression. She had never consulted a gynecologist; she either endured the pain of menstrual symptoms or, if the pain was severe, she went to a pharmacy to purchase painkillers and other medication to cope with the pain. She believed that a gynecologist only examined pregnant women and was unsure whether it would be acceptable to consult a doctor for menstruation-associated symptoms.

### 3.4. Comparison of Athletes and Non-Athletes Regarding Menstruation-Associated Symptoms

The participants were divided as follows: 136 athletes and 64 non-athletes. Pain, one week before and during menstruation, was rated from 0 for no pain at all to 10 for the most severe pain imaginable. Similar to Table 1, the athlete and non-athlete groups were classified according to the pain intensity, with participants responding 0 being classified as the no-pain group, 1–4 as the mild group, 5–7 as the moderate group, and 8–10 as the severe group.

At one week before menstruation, athletes reported no pain (44%), mild pain (36%), moderate pain (15%), or severe pain (5%). In the non-athlete group, respondents reported no pain (31%), mild pain (34%), moderate pain (23%), or severe pain (11%). The degree of pain during menstruation was none (7%), mild (34%), moderate (29%), and severe (29%) in the athlete group, and none (6%), mild (31%), moderate (31%), and severe (31%) in the non-athlete group (Table 5).

## 4. Discussion

The present study evaluated the menstrual status of female college students and clarified the differences in condition among women stratified according to the degree of their menstrual pain. It was alarming that 60% of the participants reported painful symptoms before menstruation, and 93% reported pain during menstruation. Of the respondents, 25% rated their premenstrual pain as moderate or severe; 60% reported moderate to severe pain during menstruation. Previous studies have reported an association between sleep duration and the severity of menstrual cramps as well as among menstrual irregularities, dysmenorrhea, insomnia symptoms, and poor sleep quality [4,37,38,39]. In addition, high-intensity training and mental stress can exacerbate premenstrual symptoms [40,41,42]. However, when analyzing these results as group data, it was difficult to identify the relationship between menstrual status, exercise habits, and sleep status obtained through the questionnaire-based fact-finding survey in the present study. Although it was not possible to determine whether factors such as premenstrual and menstrual pain of the participants in this study were primary or secondary, a significant correlation was observed between the degree of pain before and during menstruation. Generally, dysmenorrhea during puberty is attributed primarily to the production and release of prostaglandins from the endometrium. It has been suggested that dysmenorrhea and PMS are related to biochemical factors such as prostaglandins and to the mental stress, physical changes, and sensitivity to stress that characterize puberty [43,44]. This suggests the same possibilities in the participants of this study as in previous studies. One limitation of this study was that it was not possible to identify the presence or absence of organic abnormalities because it did not include a survey of the participant’s medical history (such as dysmenorrhea and endometriosis). The positive correlation between dysmenorrhea and premenstrual symptoms, excluding organic abnormalities, requires further detailed investigation.

Meanwhile, although the majority of respondents reported regular menstrual cycles, 48 (24%) had irregular menstrual cycles or no menstruation for ≥3 months, and 38 (19%) had excessively short (3 days or less) or long (7 days or more) menstruation in terms of the number of bleeding days. These are symptoms that may involve hormonal imbalance and poor ovarian and uterine function. Regarding sleep status, no significant difference was observed in the mean sleep duration between those with normal and poor sleep quality; however, those with poor sleep quality required over 60 min to fall asleep, and their daytime wakefulness tended to be lower. These findings clearly indicate that various factors are intricately related to both menstruation-associated symptoms and sleep quality assessment. Therefore, further research is required to attain a detailed understanding of the actual conditions, such as lifestyle factors.

Differences in physical condition according to the degree of menstrual pain were examined based on the results of the body composition analysis. The present study did not find any relationship between menstrual symptoms and body composition metrics such as BMI and bone mass. However, the findings suggested that muscle mass and fat mass exert an effect on the degree of premenstrual pain; these relationships require further investigation. PMS, including premenstrual pain, is defined as the mental and physical symptoms that follow the luteal phase and occur 3–10 days before menstruation and diminish or disappear with the onset of menstrual bleeding [45]. The type and degree of symptoms vary by individual; psychosocial and environmental factors may alter the presentation of these symptoms. High BMI, high lipid intake, nutritional status, smoking status, and stress have all been reported as potential factors in the development of PMS [46,47,48,49,50,51]. These findings suggest that understanding and improving lifestyle habits can lead to relief from premenstrual pain and other symptoms.

The case studies conducted on menstruation-associated symptoms in the present study confirmed that some respondents experienced both physical and psychological symptoms, including irregular menstrual cycles, PMS, severe menstrual cramps, and symptoms that were suspected to be dysmenorrhea. Previous studies have reported that the type and severity of menstrual complications vary between individuals [52,53]. Although the moderately small amount of data is a limitation of the present study, we reaffirm the importance of case studies for women’s health issues because some of these issues, including menstruation-associated symptoms, may be immeasurable using data from a group. The case studies mentioned in the present research mentioned the interviews with student athletes; however, even among female non-athletes, menstruation-related consultations were not uncommon. When the participants in this study were classified into the athlete and non-athlete groups, no differences in the degree of pain before and during menstruation were observed based on the level of sports activity of the participant. Ravi et al. (2021) noted that not all studies had found consistent results regarding the association between training volume and menstrual dysfunction [54]. These findings indicate that all young females, including athletes, should be knowledgeable about health issues specific to women. Although it is common for female students to have concerns about menstruation-associated symptoms, many are reluctant to visit a gynecologist. In Japan, young female students often associate visiting a gynecology clinic with feeling inadequate; however, this can lead to the neglect of concerning symptoms and a delay in disease detection. Currently, when a male gynecologist performs an internal examination, female nurses are always present, thereby ensuring that first-time female patients undergoing a medical examination are comfortable. Furthermore, some hospitals have established outpatient clinics for female athletes; these gynecology clinics have facilitated doctor consultations for several women. In fact, a female student who visited the gynecology clinic stated that she was nervous before the visit but was glad to have had the visit because the doctors and nurses were highly attentive and explained her medical condition in an easy-to-understand manner. Education about menstruation is both a sensitive topic for young women and an important aspect of their future health. To protect reproductive health and rights throughout a woman’s life, it is crucial that the youth receive menstrual education. Therefore, creating an environment where young individuals feel comfortable while discussing menstruation-associated health issues and can obtain accurate information is necessary. Schools have nurses and/or counselors who are involved in healthcare and health education as well as in the improvement of the wellbeing of children. Therefore, we propose the need to create a mechanism for information sharing, such as setting up a conference forum that includes case studies, to collaborate with school nurses and school counselors who regularly consult with children.

## 5. Conclusions

The present survey of the menstrual status, exercise habits, sleep status, and body composition of female college students revealed that 60% of the participants reported painful symptoms before menstruation, and 93% reported pain during menstruation. This includes 25% of total respondents with moderate or severe pain before menstruation and 60% with moderate or severe pain during menstruation. In addition, the study findings clarified that various factors are intricately related to both menstruation-associated symptoms and sleep quality assessment, indicating the need for further research to attain a detailed understanding of other factors, such as lifestyle and habits, in the future. The relationship between the degree of menstrual pain and body composition suggests that understanding and improving lifestyle habits can relieve premenstrual pain and other symptoms. Because no clear differences in the degree of pain before or during menstruation were observed based on the level of sports activity, being aware of one’s specific health status is important not only for athletes but for all young girls. Moreover, this study reaffirmed the importance of conducting case studies because the types and degrees of symptoms associated with menstruation vary in every individual, and some health issues specific to women may be immeasurable using data derived solely from group studies. Education about menstruation is crucial for the future health of young women. Hence, it is necessary to create an environment that encourages young women to have discussions related to menstruation-associated health issues as well as provide appropriate information regarding these issues.

## Figures and Tables

**Table 1:** Pain intensity one week before and during menstruation.

Pain Intensity	1 Week before Menstruation		Menstruation	
0	40%	(80)	7%	(14)
1	10%	(19)	7%	(13)
2	11%	(21)	12%	(23)
3	10%	(19)	7%	(14)
4	6%	(12)	8%	(16)
5	7%	(13)	6%	(12)
6	8%	(15)	9%	(17)
7	4%	(7)	16%	(31)
8	5%	(9)	14%	(27)
9	1%	(2)	9%	(17)
10	2%	(3)	8%	(16)

( ) indicates the number of people.

**Table 2:** Competition events of the participants.

Sports Competitions	*n*
Track and Field	21
Basketball	16
Softball	14
Hardball	13
Marching	12
Volleyball	11
Judo	10
Kendo	10
Dance	9
Cheerleading	8
Handball	6
Soccer	3
Golf	1
Tennis	1
Rifle Shooting	1
Athletic club	31
No affiliation	33

**Table 3:** Results of the PSQI scores.

PSQI Item	PSQI < 6	PSQI ≧ 6
Subjective sleep quality	0.96 ± 0.63	1.68 ± 0.66
Sleep latency	0.65 ± 0.75	2.05 ± 0.85
Sleep duration	0.34 ± 0.49	1.51 ± 1.00
Habitual sleep efficiency	0.12 ± 0.37	0.45 ± 0.78
Sleep disturbance	0.54 ± 0.50	0.91 ± 0.39
Use of sleep medication	0.02 ± 0.25	0.12 ± 0.47
Daytime dysfunction	0.45 ± 0.67	1.32 ± 1.05
PSQI global score	3.08 ± 1.43	8.04 ± 2.14

**Table 4:** Pain intensity at one week before menstruation and body composition.

Pain Intensity	*n*	InbodyScore		*p*-Value	FFM (kg)	*p*-Value		BFP (%)	*p*-Value		
0	56	77.9 ± 4.5	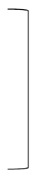	0.049	42.9 ± 5.9	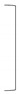	0.088	26.4 ± 5.0	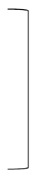		0.096
1–4	43	77.2 ± 4.8			40.1 ± 5.1			26.3 ± 5.3		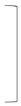	
5–7	24	75.5 ± 4.3			40.2 ± 5.9			27.6 ± 5.9			0.088
8–10	6	72.7 ± 4.7			40.8 ± 4.8			32.0 ± 4.2			

FFM: fat-free mass; BFP: body fat percentage.

**Table 5:** Pain intensity one week before menstruation and during menstruation in athletes and non-athletes.

	Athlete *n* = 136				Non-Athlete *n* = 64			
Pain Intensity	1 Week before Menstruation		Menstruation		1 Week before Menstruation		Menstruation	
0	44%	(60)	7%	(10)	31%	(20)	6%	(4)
1	9%	(12)	7%	(10)	11%	(7)	5%	(3)
2	10%	(13)	10%	(14)	13%	(8)	14%	(9)
3	12%	(16)	7%	(9)	5%	(3)	8%	(5)
4	6%	(8)	10%	(13)	6%	(4)	5%	(3)
5	4%	(6)	8%	(11)	11%	(7)	2%	(1)
6	7%	(10)	10%	(14)	8%	(5)	5%	(3)
7	3%	(4)	11%	(15)	5%	(3)	25%	(16)
8	4%	(5)	15%	(20)	6%	(4)	11%	(7)
9	0%	(0)	7%	(10)	3%	(2)	11%	(7)
10	1%	(2)	7%	(10)	2%	(1)	9%	(6)

( ) indicates the number of people.

## Data Availability

The data presented in this study are available upon request from the corresponding author. The data are not publicly available because of legal and privacy issues.
